# Long-term consumption of green tea protects the mental health of middle-aged and older adult men by improving inflammation levels

**DOI:** 10.3389/fpubh.2025.1531953

**Published:** 2025-02-25

**Authors:** Zhenyu Wan, Qirong Wan, Xucong Qin, Gaohua Wang, Hong Lin, Yong Jin, Bing Wan, Linfeng Ai, Juan Wei

**Affiliations:** ^1^Department of Psychiatry, Renmin Hospital of Wuhan University, Wuhan, Hubei, China; ^2^Yichang Mental Health Center, Yichang, Hubei, China; ^3^Department of Radiology, Affiliated Renhe Hospital, China Three Gorges University, Yichang, Hubei, China; ^4^Jingshan Psychiatric Hospital, Jingmen, Hubei, China

**Keywords:** middle-aged and older adult, depression, mental health, green tea, inflammation, hormone

## Abstract

**Background:**

Middle-aged and older adult men are at a heightened risk of depression. Green tea, as a popular beverage, has drawn widespread attention for its health benefits. However, there remains controversy over the effects of green tea on combating depression and regulating hormones.

**Objective:**

This study aimed to investigate the effects of long-term green tea consumption on depression levels, hormones, and brain structure in, middle-aged and older adult men.

**Methods:**

A total of 280 volunteers participated in the study, divided into a tea-drinking group and a control group. Basic demographic information and biological marker data, as well as MRI data from some of the volunteers, were collected. A controlled study was conducted to explore the effects of long-term tea drinking on them.

**Results:**

BMI (*p* = 0.002), depression level (*p* = 0.003), insomnia severity (*p* = 0.008), and systemic inflammation index (*p* = 0.009) were significantly lower in the tea drinking group, and their testosterone levels were significantly higher than those in the control group (*p* = 0.001). Moreover, GM volume in the right precuneus in the control group was significantly reduced compared with that in the tea drinking group.

**Conclusion:**

Long-term tea consumption helps reduce BMI and increase testosterone levels in middle-aged and older adult men, and it can also reduce their risk of depression by lowering inflammation and improving sleep quality. Additionally, long-term tea consumption may have the potential to delay brain aging in middle-aged and older adult men.

## 1 Introduction

Depression is a common mental disorder that seriously affects people’s social functioning and quality of life, with over half of all suicides being related to depression. The World Health Organization categorized severe depression as the third leading cause of global disease burden in 2008, predicting it to become the first by 2030 (1, 2). Middle-aged and older adult individuals are more susceptible to depression due to facing more diseases and neurological changes associated with aging. Among adults aged 55 and above, 2% suffer from severe depression, and 10–15% of the older adult exhibit significant depressive symptoms. Moreover, the prevalence of depression increases with age (3), with women having a higher risk but men experiencing more severe consequences (4), with a significantly higher risk of suicide than women (5). There is growing attention on how to reduce the risk of depression in middle-aged and older adult men and avoid such dire outcomes. Research suggests that physical activity can effectively improve cognitive function in middle-aged and older adult populations (6), and reduce the risk of depression through various pathways (7, 8). However, in the past 20 years, the amount of physical activity among older adult individuals in China, especially men, has significantly decreased, with more time spent on activities like watching TV and playing mahjong (9). This trend poses a risk for the onset and progression of depression. Consequently, there is an urgent need to identify and implement additional strategies to lower depression risk in this population. One such strategy is the consumption of green tea, a widely consumed, affordable, and healthy beverage, especially popular among middle-aged and older adult men in China (10, 11). Its health benefits have garnered significant attention.

Green tea contains abundant natural bioactive compounds that have been shown to improve physical function and promote metabolism (12–15). Numerous studies have also indicated that green tea may help reduce the risk of depression (16–18). However, there is still no consensus on this conclusion (19, 20), possibly due to significant differences in the observed indicators [such as the amount, concentration, and frequency of green tea consumption (21–23)] among researchers. In recent years, some animal studies have suggested that the effects of tea bioactive compounds on endocrine function may be influenced by the duration of use (24, 25). However, few studies have focused on the long-term effects of the habit of drinking tea on the human body. As a daily beverage, its long-term accumulation’s impact on the body cannot be ignored. Therefore, this study primarily aims to explore the effects of long-term green tea consumption on the levels of depression, hormones, and brain structure in middle-aged and older adult men through a comparative study.

## 2 Methods

### 2.1 Study design and sample recruiting

The study was conducted from June 2023 to March 2024 in a rural area of Hubei Province, China, known for the production of green tea (Simian tea), a non-fermented tea rich in a variety of natural active ingredients, mainly tea polyphenols, which is widely and long-term used by local residents. We randomly selected three natural villages in the area and surveyed all males in the villages. The recruitment of research subjects was overseen by the local village leaders, and questionnaire interviews and sample collection were conducted by trained psychiatric doctors and nurses. The questionnaire covered basic demographic information, levels of insomnia, and depression. Individuals who consumed green tea at least 6 days a week, with at least 1 cup (500 mL) per day, for at least 20 years, were defined as the long-term tea-drinking group, while the control group consisted of individuals who never or rarely consumed green tea (<1 time/half a year). Those meeting the inclusion criteria were required to sign an informed consent form and have blood samples collected after completing the questionnaire survey. Subsequently, a stratified random sampling method was used to select volunteers from both groups for MRI data collection. To determine the appropriate sample size for the study, we used G*Power (version 3.1), a widely recognized software tool for conducting power analyses. The calculation was based on an independent-samples *t*-test, given by the formula.


$$n1=n2=\frac{2.{\left(\sigma_{z}+\sigma_{\beta }\right)}^{2}}{\text{Cohen}\prime s\;d^{2}}\;\left(\sigma_{z}=z_{1-\alpha /2},\;\sigma_{\beta }=z_{1-\beta }\right)$$

Where the significance level (*α*) was set to 0.05, statistical power (β) was set to 0.90, and effect size (Cohen’s d) was set to = 0.5. A required sample size of 86 participants per group, with a total of 172 participants for both groups combined. This sample size provides 90% power to detect a significant difference between the groups at the specified effect size and significance level. Finally, 280 people were enrolled in the study and 71 completed MRI data collection.

Inclusion criteria were as follows: (1) meeting the corresponding inclusion criteria for the study groups; (2) voluntary participation in the research and ability to provide written informed consent; (3) participants capable of understanding the questionnaire’s questions and completing it; (4) cooperation with sample and MRI data collection; and (5) male participants aged 45 and above.

Exclusion criteria were as follows: (1) a history of psychiatric illness or a family history of psychiatric disorders; (2) recent experience of major traumatic events; (3) have serious physical illness, or have systemic lupus erythematosus, thyroid disease and other diseases that may affect hormones; (4) current or past use of psychiatric medications or hormonal drugs; and (5) recent use of coffee or energy drinks that may have an effect on hormones.

### 2.2 Variables

#### 2.2.1 Basic demographic information

The basic information includes age, height, weight, heart rate, blood pressure, marital status (married, unmarried, widowed), ethnicity, usual residence (urban or rural), education level (illiterate, primary school, junior high school and above), family annual income classified according to the Chinese household income level table into: below 10,000, 10,000–29,999, 30,000–79,999, above 80,000, religious beliefs, whether the participant is an only child, and whether they live alone. The level of social support was assessed using the Perceived Social Support Scale (PSSS), which consists of three subscales with a total of 12 items. A higher score indicates higher levels of social support, with good reliability and validity (26). The beverage consumption habits of volunteers were investigated by professional staff and recorded, including the type, frequency, time, etc.

#### 2.2.2 Sleep behavior

The Insomnia Severity Index (ISI) was used to screen the severity of participants’ insomnia. The scale consists of 7 items, with each item rated on a scale of 0–4, totaling 28 points. A higher score indicates a higher degree of insomnia. This scale has good validity and reliability for assessing insomnia severity (27, 28). As the above questionnaire cannot reflect the duration of sleep each day, we also separately recorded the volunteers’ sleep duration.

#### 2.2.3 Hormones and inflammation

Participants were asked to fast, and blood samples for further analysis were collected every morning (7–9 a.m.). Blood samples were collected using standard venipuncture in the antecubital vein, and 10 mL of blood samples from each participant were drawn into two 5 mL Vacutainer tubes for routine blood analysis and hormone assay. Platelet count, lymphocyte count, and white blood cell count in blood were measured by routine blood analysis to calculate Systemic Immune Inflammation Index (SII), which can reflect local immune response and systemic inflammation, and has been widely used in disease prediction and research (29–31). Enzyme-linked immunosorbent assays were used to analyze Thyroid-Stimulating Hormone (TSH), Free Triiodothyronine (FT3), Free Thyroxine (FT4), sex hormones (testosterone and estradiol), and inflammation markers.

#### 2.2.4 Depression level

Participants’ levels of depression were assessed using the Patient Health Questionnaire-9 (PHQ-9). This questionnaire consists of nine items, with scores ranging from 0 (not at all) to 3 (nearly every day), with higher scores indicating more severe depressive symptoms. The PHQ-9 demonstrates good sensitivity and specificity and is widely used for depression screening in primary care and research settings (32).

#### 2.2.5 MRI data acquisition

Taking into account the economic costs involved, we applied a stratified random sampling method to recruit participants for this study. Through this process, a cohort of 71 people was eventually established, of which 35 were in the tea drinking group and 36 were in the control group.

Structural MRI data was acquired on a 3-T UIH scanner (uMR 780) at Three Gorges Uni Renhe Hospital, and utilized the following parameters: repetition time (TR) = 7.2 ms; echo time (TE) = 3.1 ms; 176 slices; field of view (FOV) = 256 × 256 mm; matrix = 256 × 256.

#### 2.2.6 MRI data processing

Structural MRI data was preprocessing using FreeSurfer v7.4.0.^1^ Specifically, analysis was performed following these steps: (1) all images were inspected for data quality prior to processing; (2) skull stripping, bias field correction, and gray-white matter segmentation (33); (3) reconstruction of cortical surface models; (4) labeling of regions on the cortical surface, as well as subcortical brain structures; and (5) nonlinear registration of the cortical surface of an individual.

Then, we derived the information of cerebral cortex regions’ thickness and volume according to the default atlas, as well as the volume of subcortical regions like amygdala or putamen, to carry on the further statistical analysis.

### 2.3 Statistical analyses

#### 2.3.1 Demographic and behavioral data

Analysis was performed using SPSS Statistics 23.0. Categorical variables are represented using *N* and %, while quantitative data are represented using means and quartiles. Group differences were analyzed using chi-square tests and non-parametric tests. To verify the correlation between tea consumption and depression levels, a Spearman analysis was first conducted, including all potential factors influencing depression levels. Subsequently, ordered logistic regression analysis was employed.

To further explore the indirect effects of tea drinking on depression, path analysis was performed by structural equation model using Amos 26.0. The bootstrapping method (5,000 resamples) was employed to estimate the 95% confidence intervals (CI) of the mediators’ indirect effect. When the 95% CI did not contain zero, the indirect effect was considered statistically significant (34). The level of significance was set at 0.05, two-tailed.

Since the volunteers included in the study have consistent ethnicity, religious beliefs, and residence, they are not included in the statistical analysis. Subgroups with sample sizes that do not meet statistical requirements are merged with other groups for analysis.

#### 2.3.2 Thickness and volume

The analyses of thickness and volume were performed using Python v3.11.7. For volume, we first got standardized volume by calculating the ratio of volume to estimated total intracranial volume (eTIV). Then, the general linear model was applied to test difference between two groups on thickness and volume of cerebral cortex or subcortical regions, with age and education were included as covariates. Following the application of the Benjamini-Hochberg method for controlling the False Discovery Rate (FDR), a threshold of *p* < 0.05 is utilized to determine statistical significance.

## 3 Results

### 3.1 The characteristics and differences between the two groups on different variables

A total of 280 individuals participated in this study, with 151 individuals in the tea-drinking group. There were no significant differences between the two groups in basic sociodemographic information such as age (*p* = 0.175), only child status (*p* = 0.353), marital status (*p* = 0.375), education level (*p* = 0.508), annual income (*p* = 0.943), smoking status (*p* = 0.397), alcohol consumption (*p* = 0.318), living alone (*p* = 0.052), occupation (*p* = 0.255), and social support (*p* = 0.913). Similarly, there were no significant differences in biological information such as heart rate (*p* = 0.590), systolic pressure (SP) (*p* = 0.149), diastolic pressure (DP) (*p* = 0.167), TSH level (*p* = 0.351), FT4 level (*p* = 0.794), FT3 level (*p* = 0.934), and estradiol concentration (*p* = 0.246). However, significant differences were observed between the two groups in sleep duration (*p* = 0.031), insomnia severity (*p* = 0.008), depression severity (*p* = 0.003), body mass index (BMI) (*p* = 0.002), SII (*p* = 0.009), and testosterone concentration (*p* = 0.001) (see Tables 1, 2 for details).

**Table 1:** The characteristics and differences between the two groups on different variables (Categorical variables).

Variables	Tea group 151 (53.9%)	Non-tea group 129 (46.1%)
Only child		
Yes	135 (89.4%)	118 (91.5%)
No	16 (10.6%)	11 (8.5%)
Marital status		
Married	144 (95.4%)	121 (93.8%)
Widowed	7 (4.6%)	8 (6.2%)
Educational level		
Illiterate	12 (7.9%)	9 (7.0%)
Elementary	53 (35.1%)	56 (43.4%)
Junior high	61 (40.4%)	48 (37.2%)
Above	25 (16.6%)	16 (12.4%)
Annual income		
<10,000	62 (41.1%)	53 (41.1%)
10,000–30,000	54 (35.8%)	44 (34.1%)
>30,000	35 (23.1%)	32 (24.8%)
Smoking		
Yes	82 (54.3%)	73 (56.6%)
No	69 (45.7%)	56 (43.4%)
Drinking		
>Once a week	53 (35.1%)	47 (36.4%)
>Once a month	29 (19.2%)	33 (25.6%)
>Once every 6 months	69 (45.7%)	49 (38.0%)
Live alone		
Yes	11 (7.3%)	18 (14.0%)
No	140 (92.7%)	111 (86.0%)
Occupation		
Peasant	126 (83.4%)	114 (88.4%)
Retire	18 (11.9%)	8 (6.2%)
Unemployed	7 (4.6%)	7 (5.4%)
Sleep duration*		
>9 h	7 (4.6%)	3 (2.3%)
7–9 h	52 (34.4%)	29 (22.5%)
6–7 h	43 (28.5%)	56 (43.4%)
<6 h	49 (32.5%)	41 (31.8%)
Degree of insomnia**		
None	120 (79.5%)	81 (62.8%)
Mild	24 (15.9%)	37 (28.7%)
Moderate and above	7 (4.6%)	11 (8.5%)
Degree of social support		
High	78 (51.7%)	65 (50.4%)
Medium	63 (41.7%)	57 (44.2%)
Low	10 (6.6%)	7 (5.4%)
Degree of depression**		
None	185 (83.7%)	113 (68.5%)
Mild	21 (9.5%)	30 (18.2%)
Moderate and above	15 (6.8%)	22 (13.3%)

*** *p* < 0.001, ** *p* < 0.01, * *p* < 0.05.

**Table 2:** The characteristics and differences between the two groups on different variables (Numerical variables).

Variables	Tea group 151 (53.9%)	None-tea group 129 (46.1%)	*Z*
Age	62.9 (57.0–69.0)	61.7 (56.0–67.5)	−1.357
Heart rate	77.0 (68–84)	76.2 (70–82)	−0.539
SP	126.9 (114.0–139.0)	123.9 (110.0–135.0)	−1.444
DP	81.6 (72.0–91.0)	79.3 (70.0–86.5)	−1.383
BMI*	23.23 (21.22–25.24)	24.05 (22.90–26.18)	−3.045
TSH (mIU/L)	2.59 (1.48–3.34)	2.72 (1.69–3.44)	−0.934
FT4 (pmol/l)	18.43 (15.61–19.48)	17.99 (15.22–19.16)	−0.261
FT3 (pmol/l)	5.33 (4.93–5.75)	5.29 (4.97–5.77)	−0.083
SII**	386.06 (260.47–451.89)	435.09 (292.09–543.45)	−2.616
Estradiol (pmol/L)	162.95 (114.72–194.95)	160.64 (108.95–177.76)	−1.159
Testosterone (ng/dL) ***	574.66 (409.00–694.48)	495.89 (362.26–608.95)	−3.390

*** *p* < 0.001, ** *p* < 0.01, * *p* < 0.05.

### 3.2 Relationship between two groups and degree of depression

**Figure 1:**
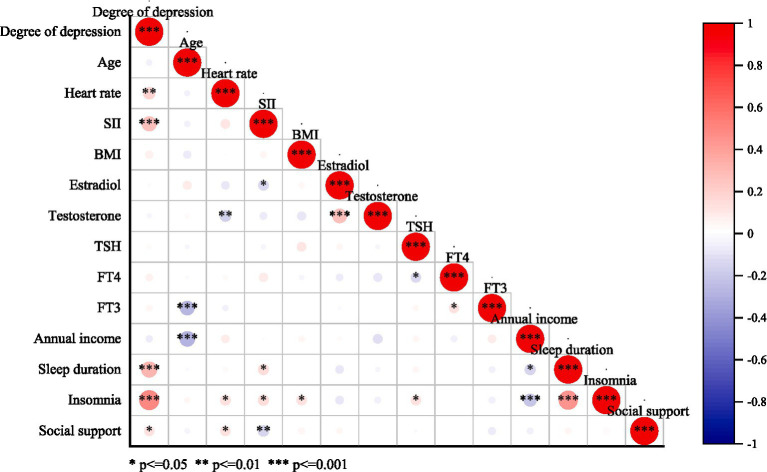
Bivariate correlation.

**Figure 2:**
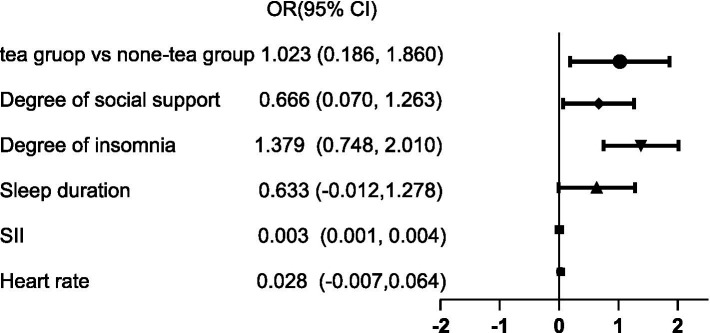
Relationship between different factors and depression degree.

First, Spearman correlation analysis was used to assess the relationship between certain biological and sociodemographic information and depression. Subsequently, factors potentially influencing depression severity were included, and ordered logistic regression was employed to explore the relationship between long-term tea drinking and depression severity. The results indicate significant correlations between systemic immune inflammation index (SII) (*p* < 0.01), heart rate (*p* < 0.01), sleep duration (*p* < 0.01), insomnia severity (*p* < 0.01), and social support (*p* < 0.05) with depression severity. Subsequent ordered logistic regression analysis revealed that the depression severity in the long-term tea-drinking group was significantly lower (*p* = 0.017) (The detailed content can be found in Figures 1, 2).

### 3.3 Path analysis model

**Figure 3:**
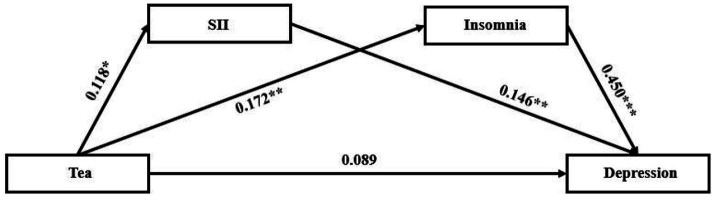
The structural equation model of the relationship between tea and depression. The value on each path indicates the standardized coefficient. SII, systemic immune inflammation. *** *p* < 0.001, ** *p* < 0.01, * *p* < 0.05.

With tea drinking as the independent variable, the degree of depression as the dependent variable, SII and the degree of insomnia as the mediating variables, structural equation model was constructed (Figure 3). The fitting indexes of the structural equation model were as follows: χ2 / df = 3.348, GFI = 0.994, RMSEA = 0.092, CFI = 0.975, AGFI = 0.941, NFI = 0.967.

The results showed that there are significant indirect effects of SII and insomnia on the relationship between tea drinking and the degree of depression (Table 3; Figure 3). The total indirect effect of tea drinking on the degree of depression was 0.103 (SE = 0.035, 95% CI [0.041, 0.180], *p* = 0.002), accounting for 51.6% of the total effect. Considering that the direct effect was not significant (0.089, SE = 0.057, 95% CI [−0.016, 0.189], *p* = 0.094), tea drinking could influence the degree of depression major by the mediating variables including SII and the degree of insomnia. The mediating effects of SII and the degree of insomnia were both significant, and were 0.019 (SE = 0.014, 95% CI [0.001, 0.059], *p* = 0.041), and 0.084 (SE = 0.032, 95% CI [0.032, 0.157], *p* = 0.003), accounted for 9.4 and 42.2% of the total effect, respectively. Figure 1 displayed the standardized path coefficients of the model.

**Table 3:** Structural equation model results for the relationship between tea drinking and depression.

Mediating effect path	Effect value	Boot SE	Boot LLCI	Boot ULCI	Relative mediation effect
Total indirect effect	0.103*	0.035	0.041	0.180	51.6%
Tea → SII → Depression	0.019*	0.014	0.001	0.059	9.4%
Tea → Insomnia → Depression	0.084*	0.032	0.032	0.157	42.2%

SE, standard errors; LLCI, lower 95% level confidence interval; ULCI, upper 95% level confidence interval; SII, systemic immune inflammation. * Indicates the indirect effect is significant (*p* < 0.05).

### 3.4 Thickness and volume

**Figure 4:**
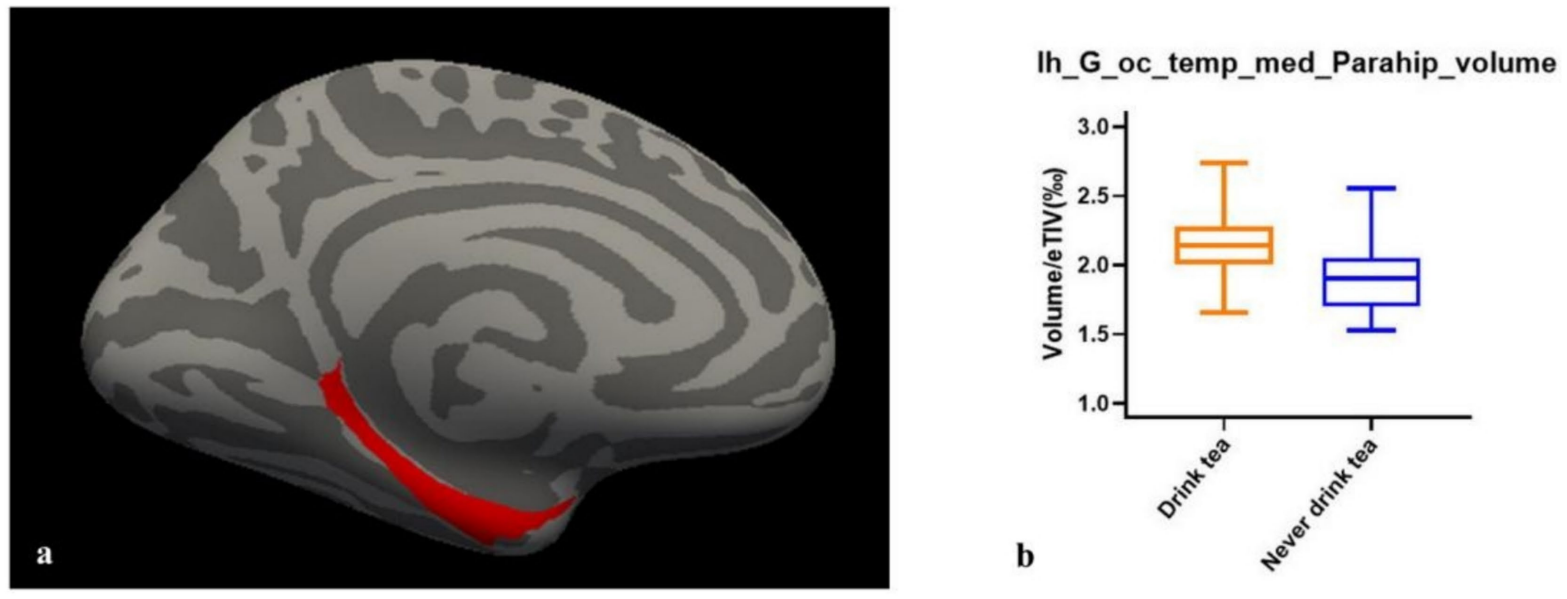
Group differences of gray matter volume in lh_G_oc_temp_med_Parahip. **(A)** Anatomical position of lh_G_oc_temp_med_Parahip. **(B)** Standardized volume (volume/eTIV) of two groups of lh_G_oc_temp_med_Parahip. lh_G_oc_temp_med_Parahip, the left medial occipito-temporal and parahippocampal gyrus. FDR *q* < 0.05.

Compared to individuals who never drink tea, tea drinkers demonstrated a higher gray matter volume in the left medial occipito-temporal and parahippocampal gyrus (lh_G_oc_temp_med_Parahip, FDR *q* = 0.01378, Figure 4). No group differences were observed in the gray matter volumes of other brain regions and subcortical structures, or in cortical thickness.

## 4 Discussion

Green tea, as a globally popular beverage, has been widely recognized for its health benefits (35). However, the research conclusions regarding the correlation between green tea and the risk of depression remain controversial, with evidence both supporting and refuting its role in reducing depression risk (19, 36). The impact on endocrine function is also subject to debate, although abundant evidence confirms the effects of bioactive compounds in green tea on various hormones (37, 38), discrepancies in research conclusions exist due to differences in experimental methods and intervention duration (24, 25, 39, 40). In everyday life, green tea is highly favored and consumed over the long term by middle-aged and older adult Chinese men (41). Therefore, this study did not consider the frequency or quantity of tea consumption as observational indicators. Instead, it emphasized the impact of long-term tea drinking as a lifestyle habit on their endocrine function, level of depression, and brain structure.

The results indicate that the testosterone concentration in individuals who consume tea over the long term is significantly higher than that in the control group. Some studies have shown that the main active compound in green tea, EGCG, can acutely inhibit basal and kinase-stimulated testosterone production (39). However, an animal study administering EGCG to rats via the jugular vein found a significant increase in plasma testosterone levels several hours later (40). These studies suggest that the effect of green tea on testosterone concentration appears to be time-dependent. This study confirms the promoting effect of long-term tea drinking on testosterone, which may contribute to improving sexual function, enhancing mood, and alleviating depressive symptoms in older adult men (42). Many studies have affirmed the influence of green tea on estradiol, suggesting that drinking green tea helps increase estradiol levels in premenopausal women (43, 44), although contradictory evidence has also been presented (45). Perhaps because the overall estradiol levels in middle-aged and older adult men are lower (46), the effect of long-term green tea consumption on it is not significant. However, the majority of related research has focused on women, and due to the existence of gender differences, more exploration is needed on the effects of green tea on male estrogen.

The concentrations of TSH, FT3, and FT4 also showed no significant differences between the two groups of people. Many animal studies suggest that active substances in green tea (mainly catechins) have a significant impact on thyroid hormones by inhibiting TPO activity, thereby reducing T3 and T4 levels and increasing TSH levels (47). However, the effects on humans may not be the same, as rodents lack high-affinity thyroxine-binding globulin, making their thyroid more susceptible to influence. Additionally, different concentrations of catechins have varying effects on the thyroid of mice (48), with only higher concentrations potentially affecting the thyroid gland and its function (49). Therefore, some researchers believe that normal consumption of green tea in humans is unlikely to lead to thyroid dysfunction (50). This study also provides evidence supporting this notion, demonstrating that long-term consumption of green tea does not significantly affect thyroid hormones in healthy middle-aged and older adult men. However, it is important to emphasize that the conclusions of animal studies should not be ignored. For populations with metabolic issues or those who consume green tea at high concentrations for an extended period, attention should still be paid to the effects of green tea on thyroid function.

Furthermore, the results indicate that long-term tea consumption significantly increases sleep duration, improves sleep quality, and reduces BMI. Green tea is rich in caffeine, which has an activating effect, and also contains theanine, which has a sleep-promoting effect (51), leading to almost contradictory effects of green tea on sleep behavior. Interestingly, green tea often has adverse effects on the sleep of individuals with initial or short-term exposure (52, 53), while older adult individuals who consume tea over the long term exhibit better sleep behavior (21). This suggests that the association between green tea and sleep behavior may be influenced by the timing of intake, and the results of this study affirm the benefits of long-term tea consumption for sleep.

The depression levels in the tea-drinking group were also significantly lower, but this conclusion faces challenges from other studies (17, 19). A follow-up study provides evidence for our conclusion. Researchers found that despite initially no difference in depression levels between the two groups, the overall depression levels in the tea-drinking group showed a more significant improvement over time (54). Similar to the impact of tea consumption on sleep mentioned earlier, the protective effect of green tea against depression may also be time-dependent, with long-term tea consumption having a more affirmative role in reducing depression risk. Additionally, pathway models indicate that tea consumption can reduce depression levels by improving sleep quality and reducing SII concentrations. There is a significant bidirectional relationship between sleep and depression (55), and the theanine in green tea can enhance sleep quality through its anti-anxiety effects (56), effectively suppressing the occurrence of chronic sleep disorders and reducing the risk of depression. The anti-inflammatory effect of green tea has been well established in many previous studies (57, 58). Inflammation is involved in the occurrence and development of almost all systemic diseases (59–62), and it is a major cause of the onset and progression of depression (63).

Some recent studies have suggested that the active compounds in green tea can inhibit the loss of hippocampal neurons in mice (64), potentially providing neuroprotective effects for older adult individuals (65, 66). Therefore, we conducted an exploratory study to compare the differences in brain structure between two groups of people. we found that the participants who never drink tea had lower GM volume in the left medial occipito-temporal and parahippocampal gyrus. Reduced volume of gray matter in wide brain regions was often regarded as the important characteristic of aging (67). Therefore, higher GM volume in this study may revealed a delay of reduced gray matter volume. The left medial occipito-temporal and parahippocampal gyrus is a set of brain regions adjacent to the hippocampus, closely associated with the limbic system. Previous studies have confirmed its relationship with memory formation and retrieval, as well as higher-order visual processing (68, 69). This may be attribute to the acts neuroprotectively of EGCG in green tea (70, 71). Our study suggests that long-term tea consumption may potentially delay brain aging in older men.

This study explored the multifaceted effects of long-term green tea consumption on middle-aged and older adult individuals. It indicated that long-term tea drinking can help reduce the BMI of middle-aged and older adult men, increase testosterone levels, and also lower the risk of depression by reducing inflammation and improving sleep quality. Furthermore, long-term tea consumption may delay brain aging in middle-aged and older adult men. However, the study’s limitations cannot be ignored: it did not use relatively precise indicators such as green tea concentration, frequency, and quantity of consumption, making it difficult to provide more accurate guidance; while the study provided evidence for the long-term benefits of tea consumption, it could not confirm how long a history of tea consumption is needed to have a significant impact on the body; moreover, the study only focused on the effects of green tea on middle-aged and older adult individuals, and as more people begin to consume green tea, a broader range of age groups should be investigated. Considering that there are large differences in hormones between middle-aged and older men and women (72), we studied the two sexes separately. The study on women can be found in another article (73). Finally, food factors and some other beverages with potential effects on hormones and inflammation were not considered in this study, and future studies need to exclude or include all foods, beverages, and dietary supplements that may affect hormones and inflammation to make the results more accurate.

## 5 Conclusion

Long-term tea consumption helps reduce BMI and increase testosterone levels in middle-aged and older adult men, and it can also reduce their risk of depression by lowering inflammation and improving sleep quality. Additionally, long-term tea consumption may have the potential to delay brain aging in middle-aged and older adult men.

## Data Availability

The original contributions presented in the study are included in the article/supplementary material, further inquiries can be directed to the corresponding author.

## Footnotes

^1^ https://surfer.nmr.mgh.harvard.edu/
